# Influence of Selected Carbon Nanostructures on the CYP2C9 Enzyme of the P450 Cytochrome

**DOI:** 10.3390/ma12244149

**Published:** 2019-12-11

**Authors:** Justyna Sekretarska, Jarosław Szczepaniak, Malwina Sosnowska, Marta Grodzik, Marta Kutwin, Mateusz Wierzbicki, Sławomir Jaworski, Jaśmina Bałaban, Karolina Daniluk, Ewa Sawosz, André Chwalibog, Barbara Strojny

**Affiliations:** 1 Department of Nanobiotechnology and Experimental Ecology, Institute of Biology, Warsaw University of Life Science, Ciszewskiego 8, 02-786 Warsaw, Poland; justyna.sekretarska@gmail.com (J.S.); jaroslaw_szczepaniak@sggw.pl (J.S.); malwina_sosnowska@sggw.pl (M.S.); marta_grodzik@sggw.pl (M.G.); marta_kutwin@sggw.pl (M.K.); mateusz_wierzbicki@sggw.pl (M.W.); slawomir_jaworski@sggw.pl (S.J.); jasmina_balaban@sggw.pl (J.B.); karolina_daniluk@sggw.pl (K.D.); ewa_sawosz@sggw.pl (E.S.); 2 Department of Veterinary and Animal Sciences, University of Copenhagen, Groennegaardsvej 3, 1870 Frederiksberg, Denmark; ach@sund.ku.dk

**Keywords:** cytochrome P450, CYP2C9, carbon nanostructures, diamond, graphite, graphene, microsomes

## Abstract

Carbon nanostructures have recently gained significant interest from scientists due to their unique physicochemical properties and low toxicity. They can accumulate in the liver, which is the main expression site of cytochrome P450 (CYP450) enzymes. These enzymes play an important role in the metabolism of exogenous compounds, such as drugs and xenobiotics. Altered activity or expression of CYP450 enzymes may lead to adverse drug effects and toxicity. The objective of this study was to evaluate the influence of three carbon nanostructures on the activity and expression at the mRNA and protein levels of CYP2C9 isoenzyme from the CYP2C subfamily: Diamond nanoparticles, graphite nanoparticles, and graphene oxide platelets. The experiments were conducted using two in vitro models. A microsome model was used to assess the influence of the three-carbon nanostructures on the activity of the CYP2C9 isoenzyme. The CYP2C9 gene expression at the mRNA and protein levels was determined using a hepatoma-derived cell line HepG2. The experiments have shown that all examined nanostructures inhibit the enzymatic activity of the studied isoenzymes. Moreover, a decrease in the expression at the mRNA and protein levels was also observed. This indicates that despite low toxicity, the nanostructures can alter the enzymatic function of CYP450 enzymes, and the molecular pathways involved in their expression.

## 1. Introduction

Carbon nanostructures have unique physical, chemical, and electrical properties that make them great candidates for various biomedical applications. Due to their structure, they can be functionalized not only with different types of chemical compounds, but also with biomolecules [1,2,3]. Therefore, in recent years, they have attracted great interest from scientists and are vastly studied in the field of biomedicine and related biological sciences for applications such as biosensors, drug and gene delivery systems, as well as tissue engineering and imaging techniques [4,5,6]. In the case of biomedical applications, it is crucial to assess the detailed impact of carbon nanostructures on cells and organs. It was reported that carbon nanostructures administered intraperitoneally can be transferred and deposited in the liver. When they have accumulated, they do not cause a toxic effect on the liver and show no effect on overall animal health status [7,8].

The liver is the main organ involved in biotransformation and the main expression site of enzymes from the cytochrome P450 family (CYP450). These enzymes constitute a large and multifunctional protein family, involved in many reactions related to the metabolism of exogenous compounds like xenobiotics and carcinogens. They also play a crucial role in the inactivation and activation of drugs [9]. CYP450 are monooxygenases, and they catalyze the incorporation of the oxygen atom to the substrate molecule with the simultaneous formation of a water molecule [10,11,12]. There are two phases of the biotransformation of drugs and xenobiotics in the human body. CYP450 enzymes play an important role in the course of the first-phase reactions. In these reactions, the introduction or unveiling of the -OH, -COOH, -SH, or -NH_2_ functional groups occurs, which in turn, leads to an increase in the hydrophilicity of the drug/xenobiotic and allows second-phase reactions and consequently, expulsion from the body [13].

One of the most important CYP450 subfamilies is the CYP2C subfamily, which metabolizes about 20% of all clinically used drugs and several endogenous compounds. Four isoenzymes are distinguished within this subfamily: 2C8, 2C9, 2C18, and 2C19 [14,15,16]. Of all the isoenzymes from the CYP2C subfamily, the CYP2C9 isoform is the most strongly expressed. It represents 50% of the entire subfamily and metabolizes about 15% of clinically used drugs, for example, non-steroidal anti-inflammatory drugs and diuretics [17,18]. As CYP enzymes are involved in the metabolism of exogenous compounds, their dysfunction may lead to slower elimination of drugs or xenobiotics from the body, thereby leading to toxicity or adverse drug reactions. The CYP2C9 enzyme predominantly metabolizes drugs such as coumarin anticoagulants, including Warfarin, acenocoumarol and, to a lesser extent, phenprocoumon [19,20]. These are drugs with narrow therapeutic indices, and therefore, there is a risk of serious bleeding if plasma drug concentrations are too high due to altered CYP2C9 activity [21].

Until now, the exact mechanisms of interaction between nanostructures and CYP enzymes have not been thoroughly researched. So far, metallic nanoparticles have been the primary targets of the investigation. It has been shown that silver nanoparticles can inhibit the most important CYP isoenzymes (including CYP2C9) in vitro, but not in vivo [22]. Zinc oxide nanoparticles have been reported to downregulate the expression of CYP1A2 but upregulate CYP2C11 and CYP3A2 isoenzymes in vivo [23]. Research evaluating the influence of polystyrene nanoparticles showed that with the reduction of size, the level of inhibition of the investigated CYP450, among them CYP2C9, increased [24]. Current scholarship suggests that nanoparticles can interfere with the activity of CYP enzymes or regulate their expression at the level of genes and proteins. However, the mechanisms of this activity are still unclear and require further research that would take into account factors such as the size, shape or surface charge of the nanoparticles [25].

The objective of this study was to investigate the influence of carbon nanostructures, such as diamond nanoparticles (DN), graphite nanoparticles (GN), and graphene oxide platelets (GO), on enzymatic activity and expression at the mRNA and protein levels of CYP2C9. Using a microsome model, we investigated the influence of these carbon nanostructures on the enzymatic activity of CYP2C9. Using hepatic-derived cell line HepG2, we have assessed the influence on gene expression at the mRNA and protein levels.

## 2. Materials and Methods

### 2.1. Nanostructures

The DN and GN produced by the explosive method were obtained from Skyspring Nanomaterials Inc. (Houston, TX, USA). The size of both ranged from 3 to 4 nm, and the purity was >95% for DN and >93% for GN. Data provided by the manufacturer show the presence of -OH, -CN, -COOH, -C-O-C, -C=O functional groups on the surface of DN. In the case of GN, manufacturer demonstrated impurities with 0.5%–1% of hydrogen, 4% oxygen, and 2% nitrogen. FTIR analysis of the used DN and GN was presented previously by Kurantowicz et al. [26]. Raman spectroscopy of the used GN was presented previously by Wierzbicki et al. [27]. XRD analysis of the used DN was presented previously by Grodzik et al. [28].

The GO produced by a modified Hummers’ method were obtained from Nanopoz (Poznan, Poland). The method was previously described by Majchrzycki et al. [29]. A typical flake diameter was 5–30 µm, and the thickness of a single layer was in the range of 0.8–1.2 nm. The GO flakes contained 35% to 49% oxygen. The elemental analysis provided by the manufacturer showed also the presence of 1%–4% of hydrogen, less than 2% of sulfur and 1% of nitrogen. Stock solutions of DN and GN at a concentration of 1000 µg/mL were prepared by suspending nanoparticle powder in ultra-pure water (MilliQ; Merck KGaA, Darmstadt, Germany), followed by dispersion using an ultrasonic bath at 550 W/m^2^ for 1 h (Sonorex Super RK 514H; Bandelin Electronic, Berlin, Germany). The GO was supplied by the manufacturer in the form of a hydrocolloid at a concentration of 4000 µg/mL. The GO stock solution (1000 μg/mL) was prepared by diluting the 4000 µg/mL hydrocolloid in ultra-pure water. Before each experiment, all stock solutions were sonicated in an ultrasonic bath for 30 min.

### 2.2. Visualization and Physicochemical Properties of Nanostructures

Transmission electron microscopy images (TEM) were performed to visualize the morphology of nanomaterials. Droplets of nanoparticles’ solutions at a concentration of 50 mg/L were placed onto Formvar-coated copper grids (Agar Scientific, Stansted, UK), and after air-drying, the grids were inspected by TEM (JEM-2000EX; JEOL, Tokyo, Japan) at 80 keV. The images were captured with a Morada 11-megapixel camera (Olympus Soft Imaging Solutions GmbH, Münster, Germany). In order to evaluate the stability of the nanoparticles’ hydrocolloids, zeta potential, and average hydrodynamic diameter measurements were performed. Measurements were conducted with a Zetasizer Nano-ZS90 analyzer (Malvern, Worcestershire, UK). Zeta potential was assessed for all nanostructures’ concentrations used in the experiments (3.13, 6.25, 50, 100 mg/L) after 120 s of stabilization at 25 °C using the micro-electrophoretic technique with the Smoluchowski approximation. The average hydrodynamic diameter of the nanostructures (using 6.25 mg/L concentration) was measured using a dynamic light scattering technique after 120 s of stabilization at 25 °C. Each measurement was repeated three times.

### 2.3. CYP450 Microsomal Model

Baculosomes^®^ is a microsomal model which facilitates the assessment of the liver CYP450 enzymes for metabolism and inhibition. The Vivid^®^ CYP450 Screening Kit with Baculosomes expressing CYP2C9 human CYP450 isoenzymes was purchased from Thermo Fisher Scientific (Waltham, MA, USA). All reagents were handled and prepared according to the manufacturer’s protocol.

Enzymatic activity in the presence of DN, GN, and GO was assessed according to the manufacturer’s protocol for the kinetic model. Hydrocolloid stock solutions were sonicated for 30 min prior to the experiment. Following the sonication, 2.5× concentrated solutions of the final concentrations (3.13, 6.25, 50, 100 mg/L) were prepared in a reaction buffer. The 2.5× solution of sulfaphenazole, a known inhibitor of the CYP2C9 isoenzyme, was also prepared. As recommended in the manufacturer’s protocol, the final concentration for sulfaphenazole was 30 µM. To avoid the inhibitory effect of the organic solvent, a 1000× higher concentration was prepared in dimethyl sulfoxide (DMSO) and diluted in ultra-pure water to obtain a 2.5× concentration. Before the experiment, a vivid substrate (BOMCC) was reconstituted in anhydrous acetonitrile. The experiment was carried out on black 96-well plates (Corning, NY, USA). In the first step, 40 µL of tested compounds, the known inhibitor (positive inhibition control) or 1× reaction buffer (control reaction) were added in triplicate to each well. In the second step, 50 µL of the Master Pre-Mix solution containing the CYP2C9 isoenzyme, the NADPH reductase, and regeneration system (glucose-6-phosphate and glucose-6-phosphate dehydrogenase), was added to each well and incubated for 10 min. Incubation allows tested compounds to interact with CYP isoenzymes. To start the reaction, a 10 µL mixture of BOMCC substrate and NADP^+^ were added to each well. Fluorescence reads were started immediately in a kinetic mode at 60-s intervals for 1 h with an excitation wavelength of 405 nm and an emission wavelength of 460 nm on the Infinite200 PRO microplate reader (Tecan Group Ltd., Männedorf, Germany). Inhibition of the reaction after 60 min was calculated from the following Equation:

$$\%\;inhibition=\left(1-\frac{X-B}{A-B}\right)\times 100\%$$

where *X* is the fluorescence intensity observed in the presence of test compound (DN, GN or GO), A is the fluorescence intensity observed in the absence of inhibitor and B is the fluorescence intensity observed in the presence of the inhibitor (sulfaphenazole).

### 2.4. Cell Culture

For cytotoxicity evaluation and gene expression at the mRNA and protein levels, the hepatocellular carcinoma HepG2 cell line was used as a model for human CYP450 enzyme expression (American Type Culture Collection, Rockville, MD, USA).

HepG2 cells were cultured in Dulbecco’s modified Eagle medium (DMEM, Gibco™; Thermo Fisher Scientific), supplemented with a 10% fetal bovine serum (FBS, Gibco™) and 1% antibiotic mix (Gibco™) of penicillin (100 U/mL) and streptomycin (100 mg/mL), and the culture was maintained at 37 °C in a humidified atmosphere containing 5% CO_2_.

For all experiments, cells were seeded at a density of 5 × 10^5^ cells/mL. For the cytotoxicity test, they were seeded on a 96-well microplate (Corning) in 100 µL of medium per well, and for the gene expression at the mRNA and protein levels, they were seeded on a six-well plate in 2 mL of medium per well. The following day, the medium was removed and replaced with fresh medium, containing dilutions of DN, GN, and GO at concentrations of 3.13, 6.25, 50, and 100 mg/L for the cytotoxicity test and 50 mg/L for the gene expression experiments. In the control group, one-tenth of the medium was also replaced with the solvent (ultra-pure water).

### 2.5. Cell Viability

Cell viability was assessed after 24 h of treatment with DN, GN, and GO with MTT assay. This colorimetric assay is based on a conversion of yellow, soluble tetrazolium salt to purple formazan crystals. The MTT solution at a concentration of 5 mg/mL was prepared by dissolving MTT powder in PBS, and 15 µL of the solution was added per each well. After 3 h of incubation at 37 °C, solubilization detergent (10% SDS, 0.01 M HCl) was added (100 µL/well). Spectrophotometer readings were performed the next day at a 570 nm wavelength on an Infinite200 PRO microplate reader (Tecan Group Ltd., Männedorf, Switzerland). Cell viability was expressed as the percentage of the control group viability, which was designated as 100%. Calculations were performed working from the following Equation:

$$cell\;viability=\frac{Abs_{test}}{Abs_{control}}\times 100\%$$

where “*Abs_test_*” is the absorbance of wells exposed to the treatment and “*Abs_control_*” is the mean absorbance of control wells.

### 2.6. Quantitative Real-Time PCR

#### 2.6.1. RNA Isolation

Total RNA was isolated with the PureLink^®^ RNA Mini Kit (ThermoFisher Scientific). After 24 h of incubation with DN, GN, and GO at the concentration of 50 mg/L, the medium was removed, and cells were washed twice with PBS and detached from plates using cell scrapers. Detached cells were collected in tubes and centrifuged for 5 min at 1200 rpm to obtain pellets, which were further re-suspended in a freshly prepared lysis buffer. Cells were homogenized using TissueLyser LT (Qiagen, Germantown, MD, USA), with a pre-frosted adapter at 50 Hz for 5 min. After homogenization, 70% ethanol was added, and the tubes were gently mixed by inverting. Further steps were performed according to the manufacturer’s protocol. The RNA concentration in each sample after isolation was measured with the NanoDrop 2000 (Thermo Fisher Scientific), and samples were kept at −80 °C.

#### 2.6.2. cDNA Synthesis

For reverse transcription, the cDNA high capacity reverse transcription kit (ThermoFisher Scientific) was used. The RNA levels in all samples were equalized, and the procedure was performed according to the manufacturer’s protocol with the following cycle conditions: 10 min at 25 °C, 120 min at 37 °C, and 5 min at 4 °C, using the 2720 Thermal Cycler (Thermo Fisher Scientific). The cDNA concentration was measured with the NanoDrop 2000 spectrophotometer, and samples were diluted to 10 ng/µL in RNase/DNase-free water. All samples were kept at −20 °C.

#### 2.6.3. Gene Expression

Gene expression at the mRNA level was determined using the ΔΔCT relative quantification real-time PCR method. The expression level of the CYP2C9 gene was assessed in reference to a housekeeping gene, GAPDH (glyceraldehyde 3-phosphate dehydrogenase). Relative gene expression (fold change [FC]) was calculated from the formula 2^−ΔΔCT^, where ΔΔCT = ΔCT of a control − ΔCT of a treated sample and ΔCT = mean CT of GAPDH − CT of a target gene.

The reaction was performed using the Step One™ Real-Time PCR System (Thermo Fisher Scientific). The thermal profile for the reaction was: 95 °C for 10 min, followed by 40 cycles of 95 °C for 15 s and 60 °C for 60 s. The reaction was set for 15 µL volume using 500 nM primer concentration of the Power SYBR™ Green PCR Master Mix (Thermo Fisher Scientific) and 50 ng of cDNA template. Details regarding the primers are shown in Table 1.

### 2.7. Western Blot

#### 2.7.1. Protein Isolation

After 24 h of incubation with the tested nanostructures, the medium was removed, and cells were washed 3× with cold PBS and detached from the wells by cell scrapers. Cells suspended in 1 mL of PBS were transferred to tubes and centrifuged at 2000 rpm for 5 min. The pellets obtained were re-suspended in a 50 µL cold RIPA buffer, containing proteases and phosphatases inhibitors. Samples were incubated at 4 °C for 30 min and vortexed every 10 min. Following the incubation, samples were centrifuged at 4 °C for 30 min at 12,000 rpm, and supernatants with isolated proteins were transferred to new tubes. The protein concentration in each sample was assessed with the Pierce™ BCA Protein Assay Kit (ThermoFisher Scientific). The assay was carried out in accordance with the manufacturer’s protocol. On the microplates (Corning), 2 µL of samples were diluted in 23 µL ultra-pure water and then 200 µL of the BCA working solution was added. Plates were incubated at 37 °C for 30 min. After incubation, an absorbance read was performed at 562 nm on an Infinite200 PRO microplate reader (Tecan Group Ltd.). The protein concentration in each sample was calculated with the formula obtained from the standard curve for known bovine serum albumin (BSA) concentrations.

#### 2.7.2. Western Blot Analysis

Samples were equalized to 25 µg/mL by dilution in ultra-pure water and a loading buffer with β-mercaptoethanol. Samples were denaturated at 95 °C for 4 min and separated by SDS-PAGE electrophoresis (5% stacking gel, 10% running gel). Electrophoretic separation was carried out at a voltage of 80 V in a stacking gel, and at 120 V in a running gel. Following the electrophoresis, a semi-dry transfer onto PVDF membrane was performed with the Trans-Blot^®^Turbo™ Transfer System (2.5 A, 7 min) (Bio-Rad, CA, USA). Membranes were incubated in 5% blotting-grade blocker (Bio-Rad) for one hour. After blocking, membranes were coated with monoclonal primary antibodies, CYP2C9 (ab4236, Abcam, UK) and GAPDH (ThermoFisher Scientific, MA, USA), and incubated at 4 °C overnight. The concentrations of antibodies were 1:1000 and 1:4000, respectively. The following day, the membranes were washed three times in a TBST buffer and incubated for one hour at room temperature with secondary antibodies: Anti-mouse (ab97020, Abcam, UK) for GAPDH and anti-rabbit (ab97048, Abcam, UK) for CYP2C9, both at a concentration of 1:5000. Prior to developing, the membranes were washed again three times in the TBST buffer. Developing was performed with CDP-Star^®^ Chemiluminescent Substrate (Merck, Germany) and Azure c400 (Azure Biosystems, CA, USA). The analysis of relative protein expression was performed with the ImageJ Fiji software [31].

### 2.8. Statistical Analysis

Cell viability data were analyzed using one-factor analysis of variance (ANOVA) with GraphPad Prism 7 (GraphPad Software, Inc., CA, USA). Differences between groups were tested with Tukey’s post-hoc test. The gene expression data were analyzed using the *t*-test. Differences at *p* < 0.05 were considered significant.

## 3. Results

### 3.1. Physicochemical Properties of DN, GN and GO

**Figure 1:**
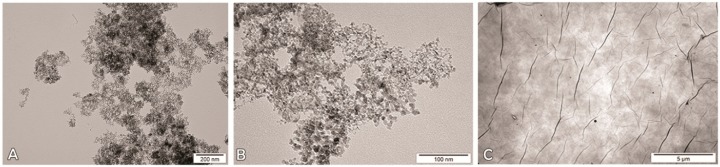
Transmission electron microscopy images of nanostructures, diamond nanoparticles (**A**), graphite nanoparticles (**B**), and graphene oxide platelets (**C**). A: Scale bar = 200 nm, B: Scale bar = 100 nm, C: Scale bar = 5 µm.

All carbon nanostructures examined showed high stability. Both DN and GO had a negative surface charge, and the value of zeta potential in all tested concentrations was >−24 mV. In comparison, GN possessed a positive surface charge, and the zeta potential values were lower, ranging from 18 to 24 mV (Table 2). The highest stability was demonstrated by GO, and the lowest stability was demonstrated by GN. Overall, all nanostructures tested had the lowest stability in the concentration of 3.13 mg/L. TEM images (Figure 1, Figures S1–S3 in the Supplementary Materials) showed that DN and GN have a tendency to agglomerate, which was confirmed by the average hydrodynamic diameter (DLS) measurements.

### 3.2. Catalytic Activity and Inhibition of the CYP2C9 Isoenzyme in the Presence of Increasing Concentrations of DN, GN and GO

**Figure 2:**
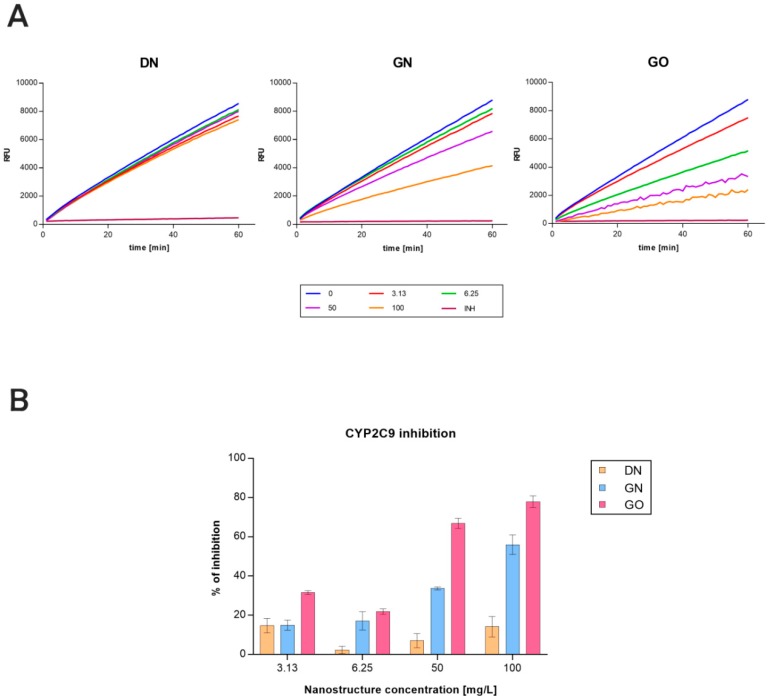
(**A**) Catalytic activity of the CYP2C9 isoenzyme in the presence of the carbon nanostructures tested, depending on concentration. Activity was measured by the enzymatic reaction product’s relative fluorescence reads (RFU—relative fluorescence units). The dark blue line depicts the control without the nanostructures (reaction not disturbed), and the brown line depicts the inhibitor control (30 µM sulfaphenazole). (**B**) Inhibition of the CYP2C9 isoenzyme after 60 min of incubation with the tested concentrations of carbon nanostructures: DN (orange bars), GN (blue bars), GO (pink bars).

The activity of the CYP2C9 isoenzyme was evaluated with an assay based on a Baculosome CYP450 model. The data collected are presented as changes in relative fluorescence of the enzymatic reaction product (Figure 2A), and as a relative inhibition with reference to a known inhibitor and an undisturbed reaction (Figure 2B).

All tested concentrations of carbon nanostructures decreased the enzymatic activity of the CYP2C9 isoenzyme. In the case of GN and GO, the inhibition rose with increasing concentrations of nanostructures. The two highest concentrations of GN and GO that were used caused inhibition to be higher than 40%. Additionally, only in the presence of GO at 50 and 100 mg/L were the disturbances in the fluorescence reads observed. The poorest inhibitory effect was noticed in the case of DN. The lowest and highest concentrations of DN used in this study showed inhibition around 15% after 60 min of the reaction.

### 3.3. Cell Viability

**Figure 3:**
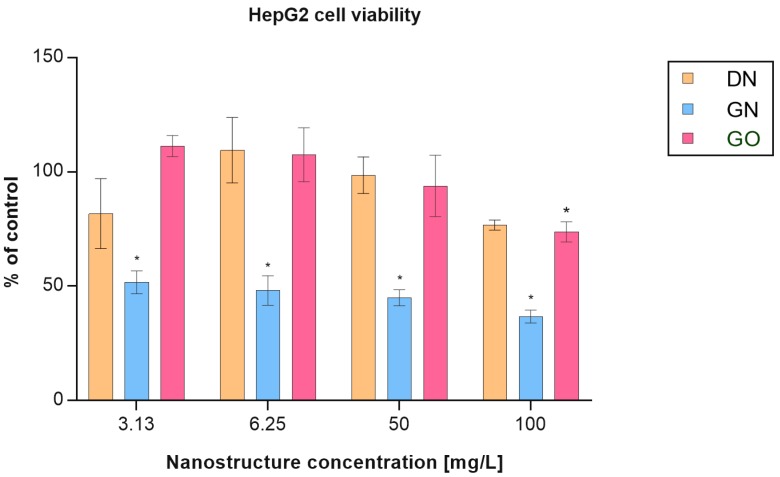
HepG2 cell viability after treatment with the tested carbon nanostructures at concentrations of 3.13, 6.25, 50 and 100 mg/L: DN (orange bars), GN (blue bars), GO (pink bars). Cell viability was determined by MTT assay. Results are presented as means with SD (n = 3) as a percentage of the control (containing only solvent in the same volume as in the nanostructure-treated wells). * *p* < 0.05 statistical significance in comparison to control (one-factor ANOVA with Tukey’s post-hoc test).

The cytotoxicity of the tested carbon nanostructures was assessed with MTT assay after 24 h of treatment. DN and GO did not decrease the viability of the HepG2 cell line. Only in the highest tested concentration (100 mg/L) was a slight decrease in viability observed (Figure 3). Each of the tested concentrations of GN caused a significant decrease in HepG2 cell viability to about 50%.

### 3.4. CYP2C9 Gene Expression at the mRNA and Protein Levels

**Figure 4:**
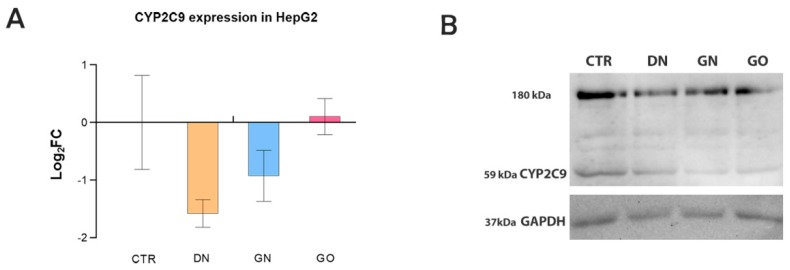
Gene expression at the mRNA and protein levels after treatment with the carbon nanostructures tested at 50 mg/L. (**A**) Relative gene expression at the mRNA level: DN (orange bars), GN (blue bars), GO (pink bars). Bars represent means with SD (n = 3, each of the biological replicates run in two technical replicates). Relative expression was calculated using GAPDH as a housekeeping gene. For easier interpretation, the results are presented as log_2_FC, where negative values indicate downregulation and positive values indicate upregulation in comparison to the gene expression in the control (untreated cells, CTR), designated as 0. Results were not statistically significant. (**B**) CYP2C9 isoenzyme expression at the protein level, control: Untreated cells (containing only solvent in the same volume as in nanostructure-treated wells), GAPDH was used as a loading control.

Real-time PCR analysis showed that both DN and GN reduced the level of mRNA of the CYP2C9 gene in the HepG2 cell line, whereas GO did not significantly affect its expression. The highest downregulation was observed in the group treated with DN: The expression was 33.4% of the control, and, in the case of GN, it was 52.5% of the control (Figure 4, Table 3). However, the differences in expression between the treated and the control group were not statistically significant (*p* > 0.05).

In the Western blot analysis, the presence of the CYP2C9 protein was demonstrated in all tested samples: a band at a height of approximately 60 kDa. Intense bands at around 180 kDa were also observed. There was a visible change in the intensity of the band at a height of approximately 60 kDa in the samples treated with DN, GN, and GO. Based on the analysis performed with ImageJ software, it was shown that the relative expression of the protein compared to expression in the control (designated as 1) in the case of ND was 0.44 (44% of control) and in the case of GN and GO, was 0.18 (18% of control).

## 4. Discussion

Intensive development of nanotechnology, including biomedical applications, leads to increased exposure to nanostructures and raises safety questions. Therefore, the possibility of adverse effects requires precise determination of the influence of nanostructures on living organisms and action mechanisms, prior to their safe application [32].

In this study, we investigated the influence of three types of carbon nanostructures: DN, GN, and GO, at high (50 and 100 mg/L) and low (3.13 and 6.25 mg/L) concentrations on the activity and expression of the CYP2C9 isoenzyme, which is one of the four isoenzymes from the subfamily CYP2C. We have compared the results obtained with the cytotoxic effect caused by the aforementioned nanostructures. Experiments were conducted using two in vitro models: The Baculosomes model (microsome model) and the HepG2 cell line model.

With the Baculosomes model, we assessed the CYP2C9 isoenzyme activity in the presence of DN, GN, and GO. Baculosomes is a model of microsomes obtained using a protein expression system based on insect cells [33]. This model allows for the evaluation of the direct interactions between the tested compounds and one specific CYP450 enzyme outside the cellular environment. Our results have shown that the CYP2C9 isoenzyme activity is inhibited in the presence of all tested nanostructures. In the case of GN and GO, concentration-dependent inhibition of enzyme activity was observed. Both GN and GO strongly inhibited the CYP2C9 isoenzyme when introduced in high concentrations of 50 and 100 mg/L. In the presence of GO at 50 and 100 mg/L, we also observed disturbances in the fluorescence reads. Such disturbances have also been noticed in our previous study investigating the CYP1A2, CYP2D6, and CYP3A4 isoenzymes [34]. In this case, the presence of GO at 50 and 100 mg/L also caused the disturbances in the fluorescent reads in all isoenzymes examined. Regarding the interactions of nanoparticles with enzymes, it is assumed that they can inhibit the enzymes by changing their conformation, disrupting their structure and/or function or blocking their active site [22,35,36]. The physicochemical properties of nanostructures, as well as environmental conditions (pH, temperature), affect their interactions with enzymes, the substrate accessibility, and their binding orientations [37]. GO platelets are rich in oxygen-containing functional groups, and therefore, they have a negative surface charge that enables them to create weak hydrogen bonds [38,39]. Zeta potential measurements confirmed a high negative surface charge of GO used in this study. In addition, various oxygen groups distributed on the surface of GO platelets provide many more interaction sites between biomolecules, leading to the formation of covalent and non-covalent bonds [40]. Moreover, nanoparticles can disrupt the structure of the cell membrane, and thus disturb the function of enzymes associated with it [41]. Cytochrome P450 enzymes belong to the group of monooxygenases that require an external electron donor [12]. In the case of microsomal CYP450 isoenzymes, the cytochrome P450 reductase is responsible for generating and transporting electrons. Transfer of the electron causes the activation of molecular oxygen and the introduction of one of its atoms into the organic substrate, with a simultaneous reduction of the second atom to the water molecule [42]. The reduced and disrupted activity of isoenzymes caused by GO may, therefore, result from the disturbance of electron transport from the cytochrome P450 reductase, which may alter the catalytic cycle of these enzymes.

In the case of DN, inhibition was not strictly correlated with the increase or decrease in concentration. Additionally, DN was characterized by the lowest ability to inhibit the activity of CYP2C9 compared to GN and GO. We have also observed that DN most strongly inhibited the activity of the examined isoenzyme in the lowest and the highest tested concentrations. The same tendency was also observed in our recent study regarding the influence of carbon nanostructures on CYP1A2, CYP2D6, and CYP3A4 isoenzymes [34]. This provides more evidence to support the assumption that with increasing concentrations, the agglomeration of DN occurs, leading to a decrease of available surface for interactions.

It was previously noticed that DN, in contrast to GN, shows no toxic effect against HepG2 cells [43]. Data collected in this study confirmed these findings. DN and GO caused no toxic effect against HepG2 cells, whereas GN significantly reduced the viability. Nevertheless, despite not causing direct toxic effects, the carbon nanostructures used in this study did alter the expression of the CYP2C9 isoenzyme at the mRNA and protein levels. This suggests that they can affect the molecular pathways and activity of biomolecules. DN and GN slightly downregulated CYP2C9 gene expression, whereas GO did not show any effect. In our previous study, GO significantly downregulated expression of the CYP1A2, CYP2D6, and CYP3A4 isoforms, but only in the HepaRG cell line [34]. Moreover, the expression of transcription mediator genes Ahr, CAR, and PXR have also been downregulated. A significantly greater reduction in the expression was observed in HepaRG cells than in HepG2. In the case of the HepaRG cell line, it has been clearly demonstrated that GO had the greatest impact on the downregulation of all isoforms. Such differences may be a result of a low expression of CYP enzymes in HepG2 compared to the HepaRG cell line [44]. The data obtained confirm that the HepG2 cell line has a low CYP450 expression and therefore is not the best model for CYP investigation.

The results obtained in this study showed that the protein level was significantly downregulated in the presence of DN, GN, and GO. Cells exposed to GN and GO had the lowest protein expression. Its relative expression was decreased by 80% compared to expression in the control. In the case of DN, the relative expression of the protein was decreased by 60%. The different impacts at the mRNA and protein levels may result from different expression regulation mechanisms. There are several mechanisms that regulate the expression of cytochrome P450 enzymes. Apart from the regulation of gene transcription, mRNA stabilization and post-translational modifications can also occur [45,46]. There is a distinct ‘ethanol-type induction’ where CYP2E1 isoenzyme expression is not altered at the mRNA level, but the protein level is upregulated through increased translational efficiency and stabilization of the protein involving ubiquitinylation and proteasomal degradation [47]. In this case, the opposite trend was observed, which may be connected with nanostructures’ tendency to attach various types of biomolecules, including proteins and lipids, which form the so-called ‘bio-corona’ [48]. Several studies reported that GO has an extremely high protein adsorption capacity [49,50,51]. It was noticed that the cytotoxic level of GO toward A549 lung carcinoma cells was greatly mitigated in the 10% fetal bovine serum. Another study had revealed that coating GO with BSA decreased cytotoxicity toward A549 cells by reducing the physical interaction of GO with the cell membrane. On the other hand, the high affinity of carbon nanostructures to bind proteins and lipids may be the reason for their disruption and decreased level in the cells [52].

Current knowledge about the influence and interactions of nanostructures with CYP450 enzymes is still very limited. Until now, only a few studies investigating the effect of carbon nanostructures on CYP450 have been carried out. It was reported that single-walled carbon nanotubes inhibit the activity and downregulate the expression of the CYP1A1 and CYP1B1 isoenzymes in HepG2 and MCF-7 cells. The authors also demonstrated that the gene expression was downregulated by blocking the binding of the AhR receptor to the enhancer region of their genes [53]. There is a need to conduct more studies regarding the influence of carbon nanostructures on CYP450 enzymes, as they play an important role in catalyzing the phase I biotransformation reactions. The reactions of this phase are a key element in the metabolism of drugs and xenobiotics [54]. Therefore, the decreased expression or activity of CYP450 enzymes, including CYP2C9, may lead to adverse drug reactions and drug toxicity [55]. The CYP2C9 isoenzyme is responsible for the metabolism of several clinically used drugs that have a narrow therapeutic index, among them mostly non-steroidal anti-inflammatory drugs, oral hypoglycemic agents, and anticoagulants, such as warfarin [56]. Warfarin, from the group of coumarin anticoagulants, is one of the most commonly prescribed drugs and one of the most important CYP2C9 substrates predominantly metabolized by this enzyme [57]. Compromised metabolism of Warfarin can result in serious health effects, as it is used to prevent thromboembolism in patients at risk [21]. Therefore, the possible influence on the activity of CYP2C9 needs to be taken into consideration when designing future biomedical applications of carbon nanostructures.

## 5. Conclusions

The results of this study have shown that DN, GN, and GO can inhibit the activity of the CYP2C9 isoenzyme and downregulate its expression at the mRNA and protein levels. This suggests that despite not causing toxic effects, carbon nanostructures can alter the molecular pathways and activities of biomolecules. This is extremely important in the case of the CYP2C9 enzyme, as it is responsible for the metabolism of several drugs with a narrow therapeutic index. This should be taken into consideration when designing biomedical applications of carbon nanostructures.

## Figures and Tables

**Table 1:** Sequence of primers used in this study.

Gene	Primer Sequence 5′→3′	PCR Product (bp)	Reference
CYP2C9	F: CTCTCTTTCCTCTGGGGCATT R: GGAAACTCTCCGTAATGGAGGTC	124	[30]
GAPDH	F: GAGAAGGCTGGGGCTCATTTG R: CATGGTTCACACCCATGACGA	97	PrimerBlast

**Table 2:** Zeta potential and average hydrodynamic diameter of examined nanostructures.

Nanostructure	Zeta Potential (mV)				Average Hydrodynamic Diameter DLS (nm)	Size TEM (nm)
Concentration (mg/L)	3.13	6.25	50	100	-	
DN	−24.27	−32.40	−27.93	−31.0	209.53	3–4
GN	18.23	22.53	21.34	24.40	619.33	4–8
GO	−48.8	−49.67	−53.87	−57.20	1747.00	>1000

**Table 3:** Detailed changes in the expression of CYP2C9 in HepG2 cells after treatment with carbon nanostructures.

Nanostructure	FC ^1^	Log_2_FC
DN	0.334	−1.580
GN	0.525	−0.929
GO	1.073	0.102

^1^ Data presented as fold change (FC), where control is designated as 1 (upregulation > 1, downregulation < 1).

## Supplementary Materials

The following are available online at https://www.mdpi.com/1996-1944/12/24/4149/s1, Figure S1: Transmission electron microscopy image of diamond nanoparticles, Figure S2: Transmission electron microscopy image of graphite, Figure S3: Transmission electron microscopy image of graphene platelets.
