# Supplementary material for: Influence of Selected Carbon Nanostructures on the CYP2C9 Enzyme of the P450 Cytochrome
Source: Materials (Basel). 2019 Dec 11;12(24):4149. doi: 10.3390/ma12244149 (PMC6947289; doi:10.3390/ma12244149)
Supplement: Supplementary file 1 [file materials-12-04149-s001.pdf]

materials

Supplementary Materials

Influence of Selected Carbon Nanostructures on the CYP2C9 Enzyme of the P450 Cytochrome

Justyna Sekretarska [1], Jarosław Szczepaniak [1], Malwina Sosnowska [1], Marta Grodzik [1], Marta Kutwin [1], Mateusz Wierzbicki [1], Sławomir Jaworski [1], Jaśmina Bałaban [1], Karolina Daniluk [1], Ewa Sawosz [1], André Chwalibog [2] and Barbara Strojny [1,*]

[1] Department of Nanobiotechnology and Experimental Ecology, Institute of Biology, Warsaw University of Life Science, Ciszewskiego 8, 02-786 Warsaw, Poland; justyna.sekretarska@gmail.com (Justyna Sekretarska); jaroslaw_szczepaniak@sggw.pl (Jarosław Szczepaniak); malwina_sosnowska@sggw.pl (M.S.); marta_grodzik@sggw.pl (M.G.); marta_kutwin@sggw.pl (M.K.); mateusz_wierzbicki@sggw.pl (M.W.); slawomir_jaworski@sggw.pl (S.J.); jasmina_balaban@sggw.pl (J.B.); karolina_daniluk@sggw.pl (K.D.); ewa_sawosz@sggw.pl (E.S.)

[2] Department of Veterinary and Animal Sciences, University of Copenhagen, Groennegaardsvej 3, 1870 Frederiksberg, Denmark; ach@sund.ku.dk (A.C.)

* Correspondence: barbara_strojny@sggw.pl; Tel.: +48-22-593-66-72

Received: 15 November 2019; Accepted: 5 December 2019; Published: 11 December 2019

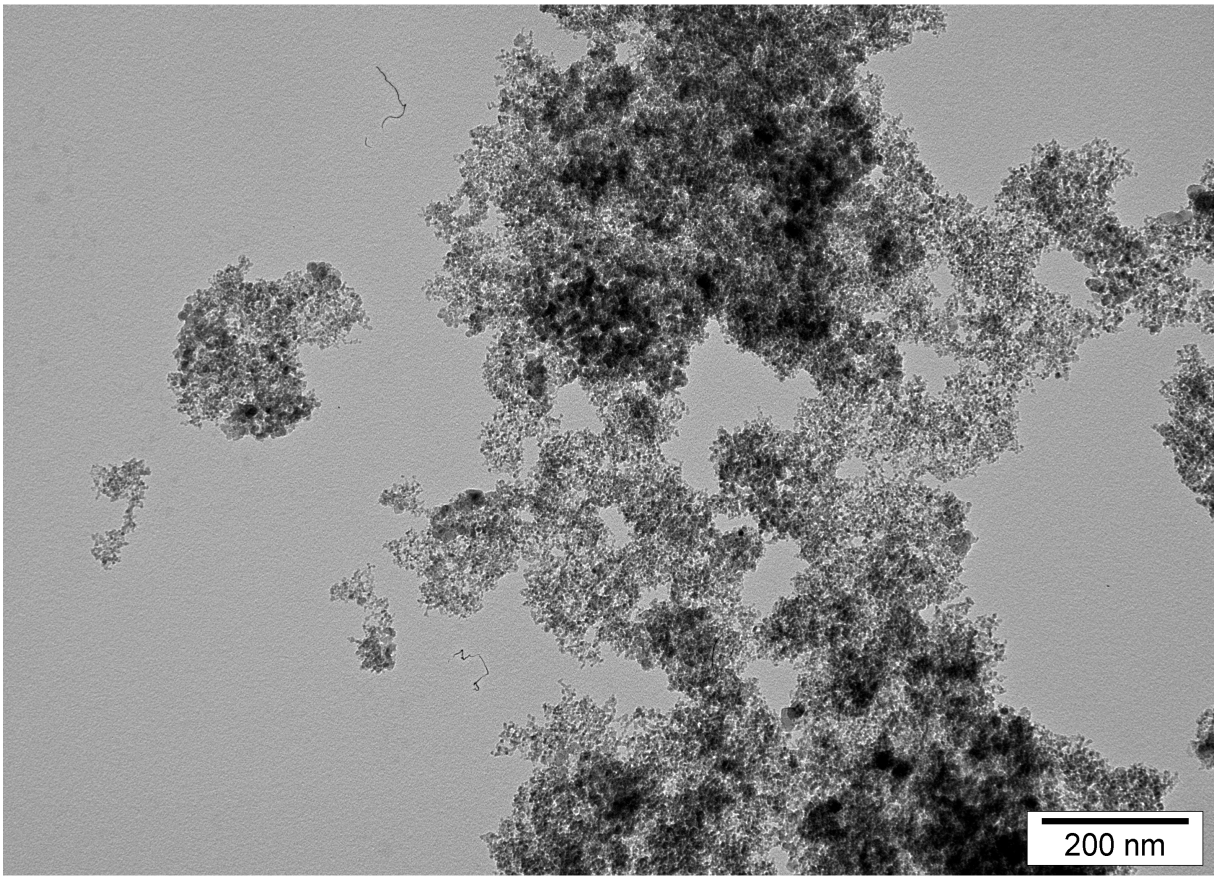

Figure S1. Transmission electron microscopy image of diamond nanoparticles, scale bar=200 nm.

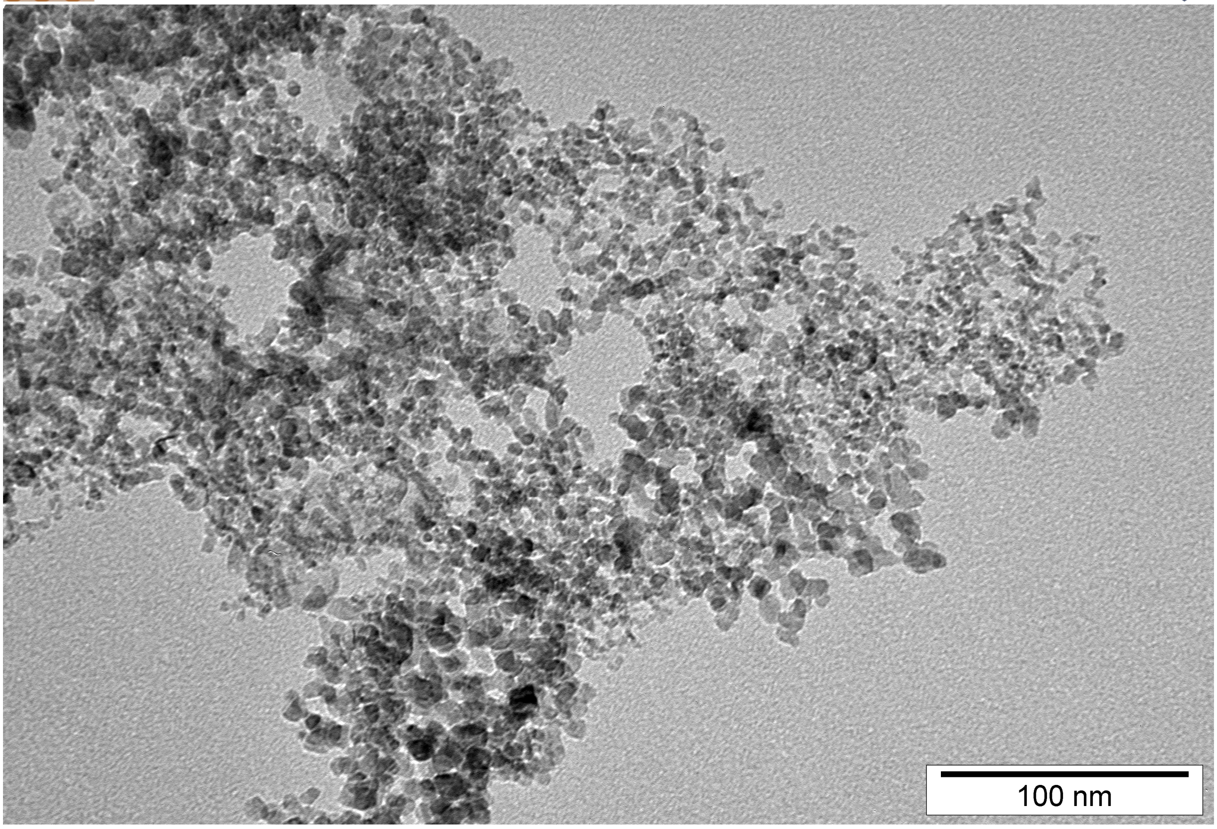

Figure S2. Transmission electron microscopy image of graphite, scale bar=100 nm.

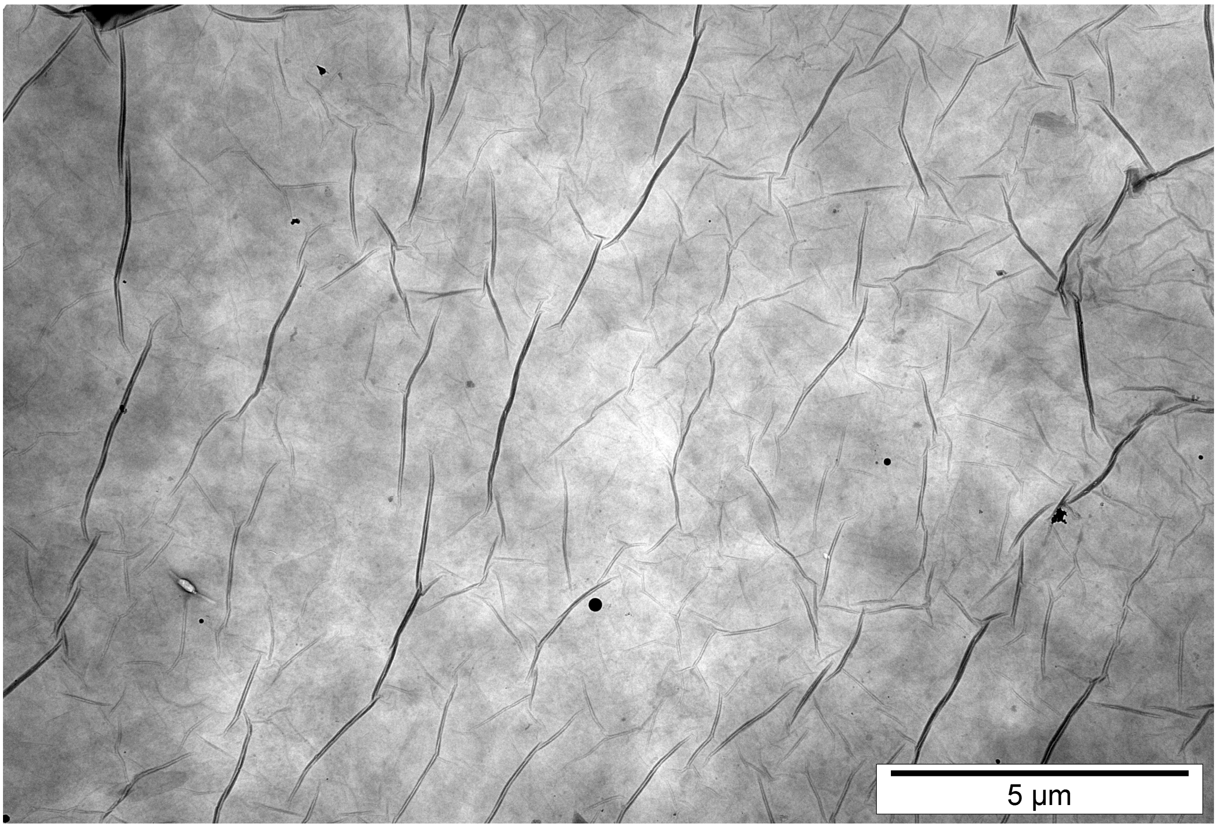

Figure S3. Transmission electron microscopy image of graphene platelets, scale bar= 5 μm.

materials

© 2019 by the authors. Licensee MDPI, Basel, Switzerland. This article is an open access article distributed under the terms and conditions of the Creative Commons Attribution (CC BY) license (http://creativecommons.org/licenses/by/4.0/).
